# Testing a candidate composite serum protein marker of skin severity in systemic sclerosis

**DOI:** 10.1093/rap/rkae039

**Published:** 2024-03-09

**Authors:** Elen Roblin, Kristina E N Clark, Claire Beesley, Voon H Ong, Christopher P Denton

**Affiliations:** Department of Rheumatology, Royal Free Hospital, London, UK; Centre for Rheumatology, University College London, London, UK; Centre for Rheumatology, University College London, London, UK; Centre for Rheumatology, University College London, London, UK; Centre for Rheumatology, University College London, London, UK

**Keywords:** systemic sclerosis, scleroderma, modified Rodnan Skin Score, biomarker, proteomics

## Abstract

**Objectives:**

Using an integrated multi-omic analysis, we previously derived a candidate marker that estimates the modified Rodnan Skin Score (mRSS) and thus the severity of skin involvement in SSc. In the present study we explore technical and biological validation of this composite marker in a well-characterized cohort of SSc patients.

**Methods:**

Cartilage oligomeric matrix protein (COMP), collagen type IV (COL4A1), tenascin-C (TNC) and spondin-1 (SPON1) were examined in serum samples from two independent cohorts of patients with dcSSc. The BIOlogical Phenotyping of diffuse SYstemic sclerosis cohort had previously been used to derive the composite marker and Molecular Determinants to Improve Scleroderma (SSc) treatment (MODERNISE) was a novel validation cohort. Multiple regression analysis derived a formula to predict the mRSS based on serum ELISA protein concentration.

**Results:**

The serum concentration of two of the proteins—COMP and TNC—positively correlated with the mRSS, particularly in early dcSSc patients. Interpretable data could not be obtained for SPON1 due to technical limitations of the ELISA. COL4A1 showed a correlation with disease duration but not overall mRSS. Patients receiving MMF showed lower serum concentrations of COMP, COL4A1 and TNC and a lower composite biomarker score not established on treatment. A revised ELISA-based three-protein composite formula was derived for future validation studies.

**Conclusions:**

Although more validation is required, our findings represent a further step towards a composite serum protein assay to assess skin severity in SSc. Future work will establish its utility as a predictive or prognostic biomarker.

Key messages

- Development of a composite blood biomarker reflecting skin thickness severity (mRSS) would be valuable for patient treatment.
- We have partially validated a previous score using quantitative serum ELISA.
- Future work is needed to further validate and refine this promising simple composite serum assay.

## Introduction

SSc is a complex multicompartment connective tissue disease characterized by fibrosis, vasculopathy and inflammation. Patients with dcSSc have a higher prevalence of life-threatening complications, most frequently related to the lung, kidney and heart [1, 2]. Symptoms relating to skin disease such as pruritis, pain and inability to perform previously easy tasks negatively impact quality of life [3]. Greater severity of skin involvement in the early stages of disease has been found to be predictive of cardiac involvement [4], scleroderma renal crisis [5], decline in lung function [6] and mortality [7, 8]. Equally, improvement in skin involvement is associated with better survival and lesser involvement of internal organ complications [9, 10].

Quantification of SSc skin disease by the modified Rodnan Skin Score (mRSS) has proved feasible in clinical trials and practice but requires expert training and regular practice to ensure reliability [11, 12]. A more objective measure of skin severity would be highly desirable, and development of a blood biomarker would be valuable to address these points.

Multilevel and high-dimensional analysis derived a composite biomarker for mRSS using the BIOlogical Phenotyping of diffuse SYstemic sclerosis (BIOPSY) cohort [13]. This identified four blood proteins that independently correlated with mRSS and also with skin gene and dermal blister expression at a separate 12-month time point. These proteins are cartilage oligomeric matrix protein (COMP), collagen type IV (COL4A1), tenascin-C (TNC) and spondin-1 (SPON1). COMP is a TGF-β-regulated matricellular protein that contributes to the integrity of the fibrillar collagen extracellular network [14]. It has been previously found to be upregulated by fibroblasts in SSc patients [15]. COL4A1 is implicated in angiogenesis and found at the dermo-epidermal junction in skin. Elevated serum COL4A1 has been positively correlated with mRSS in early dcSSc [16]. TNC perpetuates the damage-associated molecular patterns that induce the differentiation of resident fibroblasts into myofibroblasts, promoting a profibrotic state [17]. It has been found to be one of the most highly upregulated extracellular matrix proteins in SSc skin biopsies [18]. SPON1 is a protein that is coded by the *SPON1* gene. It acts as an adhesion molecule in the basement membrane and has previously been found to be upregulated in the sera of SSc patients [19]. Taken together, these proteins likely reflect overlapping aspects of SSc skin pathobiology that are not overly influenced by other disease compartments. To further develop and validate this composite marker, we measured the same analytes in serum by commercial ELISA kits.

## Methods

### Patient and control samples

Sera were obtained from dcSSc patients included in the previously described BIOPSY cohort and a new validation cohort: Molecular Determinants to Improve Scleroderma (SSc) treatment (MODERNISE). All samples were collected after written informed patient consent. Collected sera were aliquoted and stored at −80°C. Subject characteristics are fully described in the Results section below.

### ELISA

Concentrations of candidate proteins were determined using commercial ELISA kits from Cusabio (Houston, TX, USA) for COL4A1 (CSB-EL005741HU: 1:100 dilution) and SPON1 (CSB-EL022599HU: no dilution), R&D Systems (Minneapolis, MN, USA) for COMP (DCMP0: 1:100 dilution), Abcam (Cambridge, UK) for TNC (ab213831: 1:100 dilution) and Biorbyt (Durham, NC, USA) for SPON1 (orb405359: no dilution).

### Statistical analysis

Simple linear regression was used for cross-sectional correlations between mRSS, disease duration and protein serum concentration. Student’s unpaired *t*-test was used to determine the difference between protein serum concentration of SSc patients receiving the standard of care (SOC) *vs* those that were not (non-SOC). After normalizing ELISA data, multiple linear regression analysis was performed to derive a formula to predict mRSS. Conformity was determined using Bland–Altman plots. Ordinary one-way analysis of variance was used for cross-sectional analysis of MMF duration and protein serum concentration.

### Ethics approval

This project was conducted in compliance with the Declaration of Helsinki and was approved by the London-Hampstead NRES Committee (MREC Reference ID 6398) for BIOPSY and London‐Fulham Research Ethics Committees (IRAS project ID 279682) for MODERNISE sample and data collection and analysis.

## Results

### Patient demographic and clinical characteristics

Table 1 summarizes clinical and demographic characteristics of the BIOPSY and MODERNISE cohorts. The BIOPSY cohort samples included 33 dcSSc patients: 23 (70%) were female; median disease duration was 6.4 years [interquartile range (IQR) 1.4–6.4]; median baseline mRSS was 16.4 (IQR 9–22.5); 31 (94%) patients harboured ANAs, of whom 10 (30%) had anti-topoisomerase antibodies (ATAs), 12 (36%) had anti-RNA polymerase III antibodies (ARAs) and 9 (27%) had other antibodies. No SSc-related deaths occurred during follow-up. A total of 17 (52%) patients were taking MMF at the time of serum sampling.

**Table 1.** Demographics of participants included in the ELISA analysis

Characteristics	BIOPSY cohort (*n* = 33)	MODERNISE cohort (*n* = 37)	Combined early dcSSc cohort (*n* = 36)
Female, *n* (%)	23 (70)	28 (76)	25 (69)
Age (years), median (IQR)	51.6 (35.0–65.6)	53.3 (39.3–53.2)	58.9 (43.2–70.3)
Disease duration (years), median (IQR)	6.4 (1.4–6.4)	9.5 (4–12.5)	2.5 (1.3–3.8)
mRSS, median (IQR)	16.4 (9–22.5)	16.9 (10.5–21.5)	20.7 (11–30)
Antibody, *n* (%)			
ATA	10 (30)	16 (43)	14 (39)
ARA	12 (36)	15 (41)	14 (39)
Anti-U3RNP antibody	5 (15)	3 (8)	4 (11)
Anti-centromere antibody	0 (0)	1 (2)	0 (0)
ANA negative	2 (6)	2 (5)	2 (6)
Other	9 (27)	4 (11)	2 (6)
Organ involvement, *n* (%)			
Lung	13 (39)	18 (49)	13 (36)
Kidney	3 (9)	3 (8)	4 (11)
Pulmonary arterial hypertension	1 (3)	4 (11)	0 (0)
Cardiac	3 (9)	2 (5)	2 (6)
Gastrointestinal	2 (6)	1 (2)	1 (3)
Overlap conditions, *n* (%)			
Rheumatoid arthritis	1 (3)	1 (2)	1 (3)
Polymyositis or dermatomyositis	6 (18)	1 (2)	4 (11)
Immunosuppression at time of sample collection, *n* (%)			
MMF	17 (52)	27 (73)	20 (56)
Methotrexate	8 (24)	8 (22)	7 (19)
Steroids	10 (30)	12 (32)	10 (28)
Tocilizumab	1 (3)	1 (2)	1 (3)
Cyclophosphamide	1 (3)	4 (11)	2 (6)
Rituximab	0 (0)	3 (8)	1 (3)
Untreated	6 (18)	3 (8)	3 (8)

The MODERNISE cohort included 37 dcSSc patients: 28 (76%) were female; median disease duration was 9.5 years (IQR 39.3–53.2); median baseline mRSS was 16.9 (IQR 10.5–21.5); 35 (95%) harboured ANAs, of whom 16 (43%) had anti-ATAs, 15 (41%) had anti-ARAs and 6 (16%) had other antibodies. One SSc-related death occurred during follow-up. A total of 27 (73%) were taking MMF at the time of serum sampling.

The early dcSSc cohort was comprised of 26 patients, 14 of whom were from the MODERNISE cohort and 22 patients of whom were from the BIOPSY cohort. Three early dcSSc patients from the MODERNISE cohort and 13 patients from the BIOPSY cohort were MMF naïve at the time of serum sampling.

For benchmarking purposes, three healthy control serum samples were also included in the ELISA measurements, with an average age of 55 years and 2 of whom were female.

Following independent analysis, a combined group of early dcSSc patients with a disease duration of <5 years from both cohorts was analysed as a third group to confirm and extend data from the two independent cohorts. Patients were designated as non-SOC if they had either never been exposed to MMF or had taken it for <6 months. Patients were designated as SOC if they had received treatment with MMF for at least 6 months prior to sample collection.

### ELISA measurements

#### COMP

**Figure 1.**
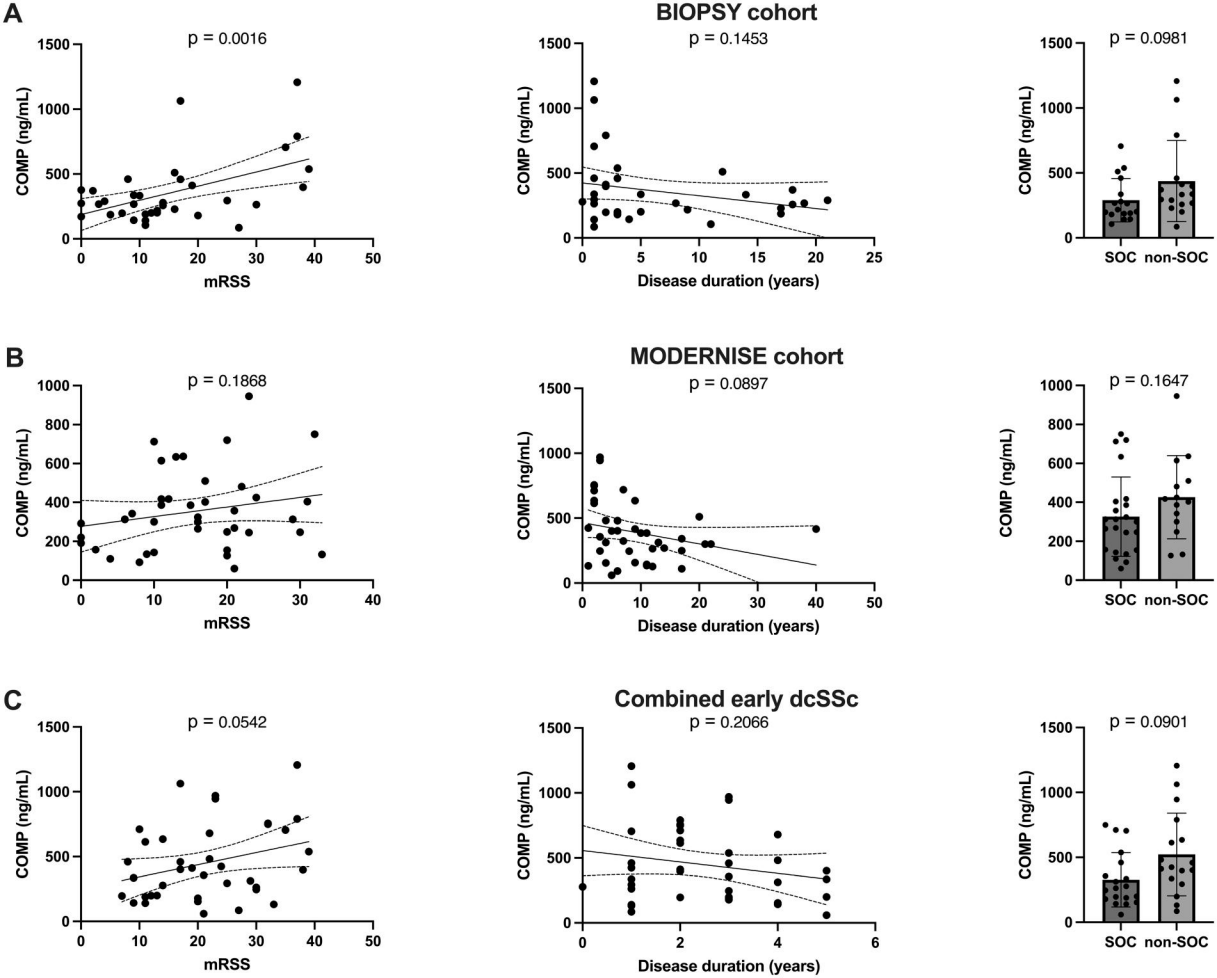
Testing and validation of COMP. COMP concentration against mRSS, disease duration and SOC in (**A**) the BIOPSY cohort, (**B**) the MODERNISE cohort and (**C**) the combined early dcSSc patients from both cohorts

In the dcSSc patients from the BIOPSY cohort, a statistically significant positive correlation was observed between serum COMP concentration and mRSS (*r* = 0.26, *P* = 0.0016) (Fig. 1A). A similar but weaker correlation was found in the MODERNISE cohort (*r* = 0.05, *P* = 0.19) (Fig. 1B). Among early dcSSc patients, there was a near statistically significant correlation between COMP concentration and mRSS (*r* = 0.097, *P* = 0.05) (Fig. 1C). In both cohorts, there was a negative correlation between serum COMP concentration and disease duration (*r* = 0.07, *P* = 0.15; *r* = 0.07, *P* = 0.09). Furthermore, SOC patients exhibited a lower COMP concentration compared with non-SOC patients in both cohorts. This difference was most pronounced among early dcSSc patients (327.6 ng/ml *vs* 522.5 ng/ml, *P* = 0.09).

#### COL4A1

In the dcSSc patients from the BIOPSY cohort (Supplementary Fig. S1A, available at *Rheumatology Advances in Practice* online) and combined early dcSSc cohort (Supplementary Fig. S1C, available at *Rheumatology Advances in Practice* online), serum COL4A1 concentration did not correlate with mRSS (*r* = 0.02, *P* = 0.46; *r* = 0.03, *P* = 0.34). The trend for correlation in the BIOPSY cohort was strengthened when isolated to non-SOC patients with a disease duration of <5 years (*r* = 0.25, *P* = 0.14) (Supplementary Fig. S2A, available at *Rheumatology Advances in Practice* online).

In the BIOPSY cohort, serum COL4A1 concentration significantly positively correlated with disease duration (*r* = 0.15, *P* = 0.03) (Supplementary Fig. S1A, available at *Rheumatology Advances in Practice* online), but for the BIOPSY cohort patients on SOC with a disease duration of <5 years there was a negative correlation (*r* = 0.24, *P* = 0.10) (Supplementary Fig. S2B, available at *Rheumatology Advances in Practice* online). A similar trend was observed in the combined early dcSSc cohort (*r* = 0.04, *P* = 0.23) (Supplementary Fig. S1C, available at *Rheumatology Advances in Practice* online), suggesting that disease duration and SOC treatment may influence the relationship between COL4A1 and mRSS. Non-SOC early dcSSc patients demonstrated numerically higher serum COL4A1 concentrations compared with those on SOC treatment in the combined early dcSSc cohort (80.7 ng/ml *vs* 56.5 ng/ml, *P* = 0.30) (Supplementary Fig. S1C, available at *Rheumatology Advances in Practice* online).

#### TNC

**Figure 2.**
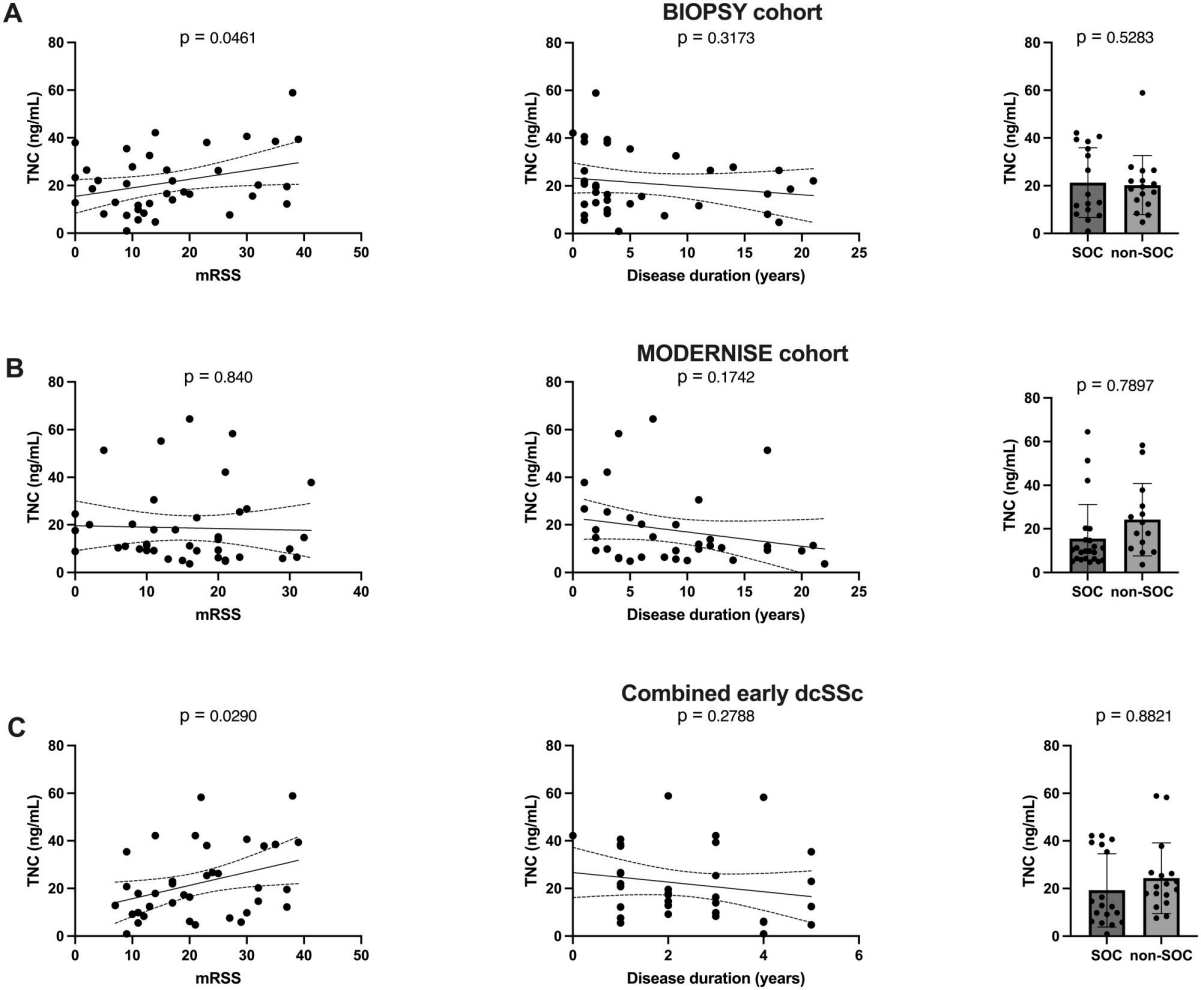
Testing and validation of TNC. TNC concentration against mRSS, disease duration and standard of care in (**A**) the BIOPSY cohort, (**B**) the MODERNISE cohort and (**C**) the combined early dcSSc patients from both cohorts

Serum TNC concentration significantly increased with mRSS in the BIOPSY cohort and among early dcSSc patients (*r* = 0.1, *P* = 0.05; *r* = 0.13, *P* = 0.03) (Fig. 2A, C). There was no association with mRSS in the overall MODERNISE cohort (Fig. 2B). Serum TNC concentration demonstrated a weak trend toward decreased serum concentration with disease duration in all cohorts (*r* = 0.03, *P* = 0.32; *r* = 0.05, *P* = 0.17; *r* = 0.04, *P* = 0.28). Serum TNC concentration was numerically higher among non-SOC patients in the MODERNISE cohort and among early dcSSc patients (15.5 ng/ml *vs* 24.25 ng/ml, *P* = 0.79; 19.28 ng/ml *vs* 24.36 ng/ml, *P* = 0.88) (Fig. 2B, C).

#### SPON1

Levels of SPON1 were below the assay range in four samples using the ELISA. There was no correlation between serum SPON1 concentration and mRSS or SOC status. There was a slight negative trend between serum SPON1 concentration and disease duration in the BIOPSY and MODERNISE cohorts (*r* = 0.02, *P* = 0.38; *r* = 0.06, *P* = 0.18) (Supplementary Fig. S3, available at *Rheumatology Advances in Practice* online). Considering the suboptimal performance of SPON1, we pursued an alternative commercial ELISA kit to reassess its suitability using samples from the BIOPSY cohort. During repeat testing, we encountered challenges in obtaining readouts for 20 samples due to low serum concentration of SPON1. Among the samples analysed, a negative association was observed between SPON1 concentration and mRSS, accompanied by a positive correlation with disease duration (Supplementary Fig. S4, available at *Rheumatology Advances in Practice* online).

### Integrated analysis of multiple analytes

**Figure 3.**
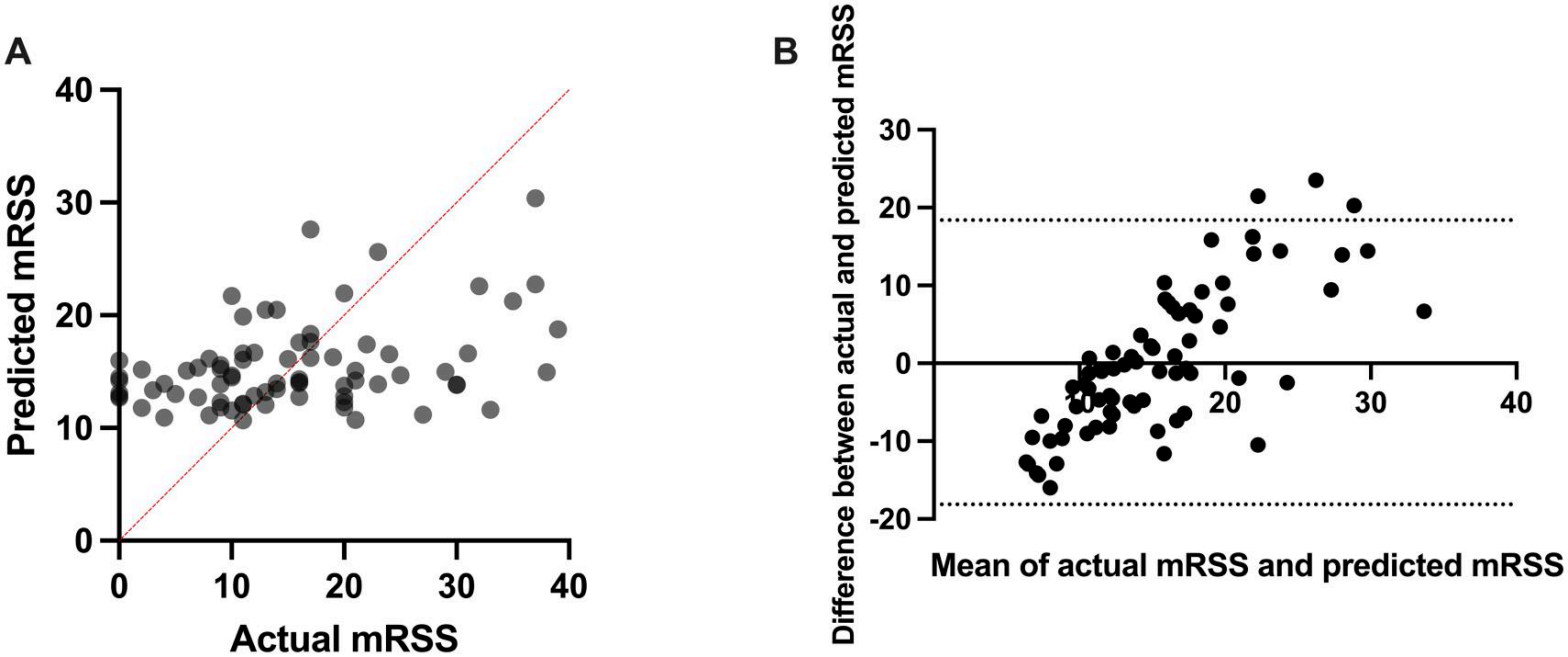
Integrated analysis of multiple analytes. (**A**) Multiple linear regression analysis and (**B**) Bland–Altman plot of predicted and actual mRSS based on predictive model from COMP, COL4A1 and TNC

We performed multiple linear regression on serum concentration of COMP, COL4A1 and TNC to predict mRSS (Fig. 3):

mRSS=9.896 + 0.01719(COMP) − 0.006481(COL4A1)− 0.002318(TNC).

Tabulated analysis of predictor variables can be seen in Supplementary Table S1, available at *Rheumatology Advances in Practice* online. The ordinary least squares regression model showed this to be significant with *r*= 0.15 and *P* = 0.009. Only COMP contributed to the regression equation with statistical significance. The Bland–Altman plot shows better conformity of results for mRSS between 10 and 20 with limits of agreement from −18.13 to 18.39.

### Effect of MMF duration on serum protein concentration and composite biomarker score

**Figure 4.**
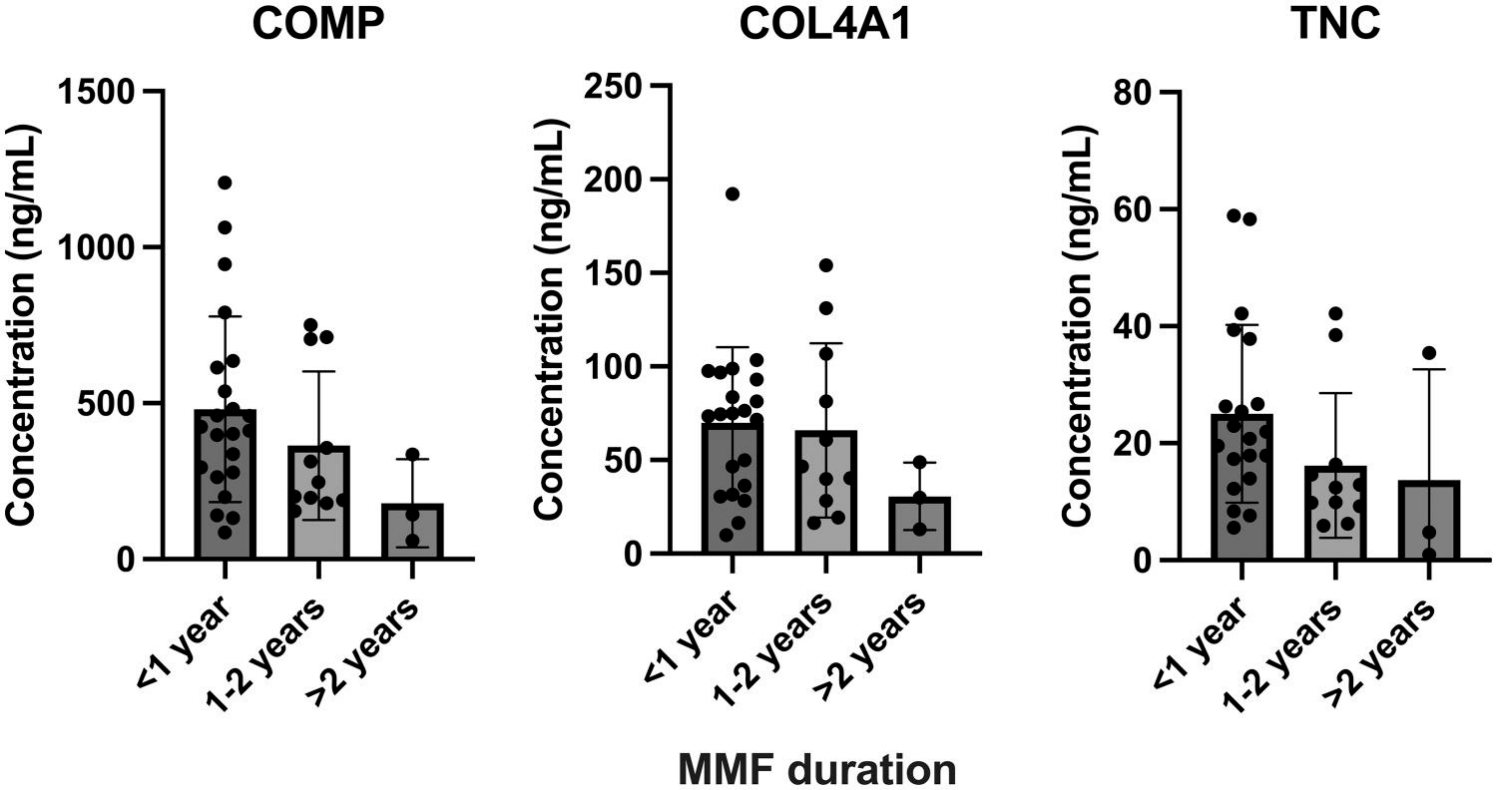
Effect of MMF treatment duration on serum COMP, COL4A1 and TNC concentrations in the combined early dcSSc cohort

Early dcSSc patients established on MMF treatment demonstrated lower levels of COMP (*P* = 0.16), COL4A1 (*P* = 0.32) and TNC (*P* = 0.15) compared with those who were either MMF naïve or had been on MMF for <1 year (Fig. 4). Early dcSSc patients with either ATA or ARA positivity demonstrated similar reductions in serum COMP, COL4A1 and TNC concentrations with increasing MMF exposure (COMP, *P* = 0.43 *vs P* = 0.59; COL4A1, *P* = 0.45 *vs P* = 0.11; TNC, *P* = 0.09 *vs P* = 0.06) (Supplementary Fig. S5, available at *Rheumatology Advances in Practice* online).

**Figure 5.**
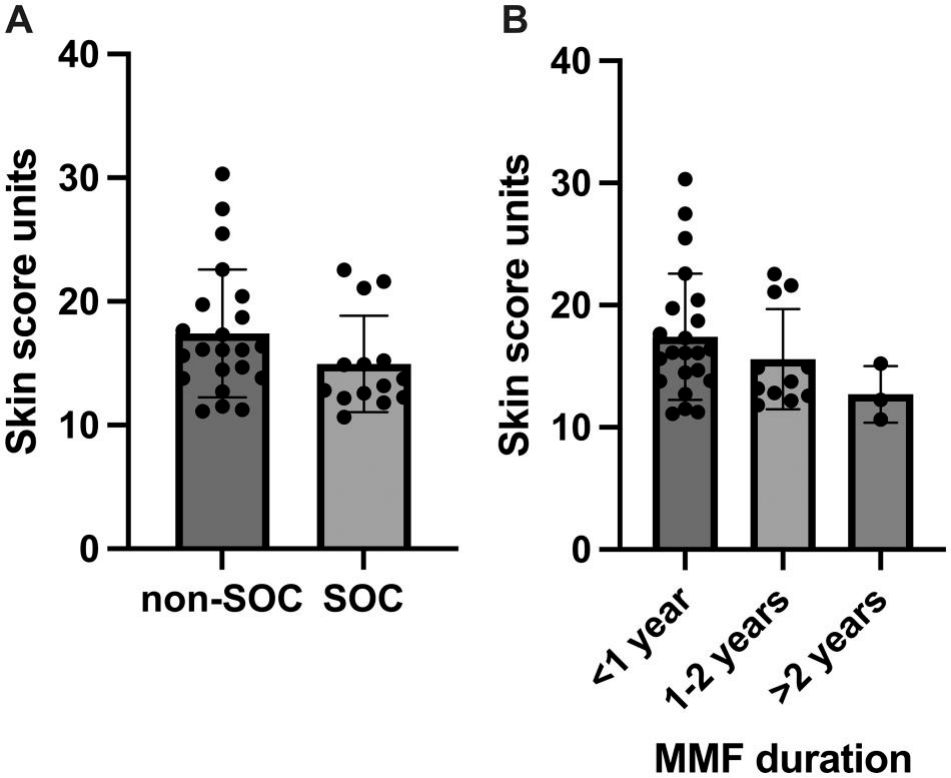
Effect of treatment and MMF duration on the composite serum biomarker score. (**A**) Composite biomarker score against the SOC and (**B**) MMF duration in the combined early dcSSc cohort

Early dcSSc patients established on SOC had a numerically lower composite biomarker than those who were not (17.4 *vs* 15.0, *P* = 0.29) (Fig. 5A). There was also a trend of lower composite biomarker score with increasing MMF duration (*P* = 0.22) (Fig. 5B).

## Discussion

We performed a validation study to explore the performance of a novel composite biomarker for prediction of mRSS in SSc using quantitative serum ELISA methodology. This was undertaken in an independent external validation cohort (MODERNISE) as well as subjects included in a previous derivation cohort (BIOPSY) [13]. We derived a formula based on absolute values that could be further tested and validated in cross-sectional studies to determine its prognostic and predictive capability as well as longitudinal studies to determine whether it is pharmacodynamic. We hope such a tool could be used in the future for stratification, subset analysis and outcome assessment. The ability to highlight patients who are more likely to develop severe disease would allow earlier and more intensive intervention and may also encourage consideration of higher-risk interventions such as haematopoietic stem cell transplantation.

A blood biomarker would allow for the development of more specific inclusion criteria for trials, focusing on patients who are likely to have progressive disease and helping to differentiate novel treatment effect from that due to background SOC immunosuppression such as MMF. A few recent trials, including the phase 3 randomized placebo-controlled trial of tocilizumab, failed to meet their primary endpoint of reduction in mRSS but did reach their secondary endpoints [20]. This might suggest that the mRSS alone is not capturing clinical improvement in the same way as other quantifiable measures such as forced vital capacity or the HAQ Disability Index [3]. A composite blood biomarker could complement mRSS as a measure of skin activity in the future.

In general, for two proteins (COMP and TNC), data were congruent. However, there was greater variability among ELISA results than those obtained using the proximity extension assay Olink platform for derivation, which has technical superiority and a larger dynamic range than ELISA.

The COL4A1 protein showed variability in serum concentration with mRSS and disease duration but did demonstrate a reduction with MMF treatment. When isolated to patients of shorter disease duration who were not on MMF in the BIOPSY cohort, there was a positive correlation between mRSS and COL4A1 serum concentration. In BIOPSY cohort patients on MMF, COL4A1 serum concentration decreased with disease duration in the first 5 years of disease. Taken together, these findings could suggest that COL4A1 is more influenced by MMF than the other investigated proteins and could explain the observed variability when looking at the whole cohort. This warrants further exploration in the future.

Interpretable data could not be obtained for the SPON1 protein. This may reflect technical limitations of the ELISA or that the dynamic range of the Olink assay was much better suited to the analysis. This can be revisited in future analyses to determine whether SPON1 may add value. Operationally, our results suggest that an ELISA-based three-protein marker excluding SPON1 may warrant further evaluation in a larger SSc cohort to better define the relationship with mRSS in early disease and the impact of standard immunosuppression.

Performance was most congruent with our previous findings of an association between serum analyte and mRSS in the combined early dcSSc cohort from the MODERNISE and BIOPSY cohorts, suggesting that this may be the most appropriate group for further validation of the biomarker. It is notable that for the plasma proteins in our previous study of the complete BIOPSY cohort, it appeared that early-stage dcSSc had the strongest contribution to statistical association in deriving the composite biomarker of skin severity. It is possible that the impact of disease duration and SOC immunosuppression is less prominent in this early dcSSc subgroup.

Treatment effect needs to be considered in interpreting our findings, especially with recent studies pointing towards significant benefit from the use of SOC immunosuppression such as MMF [21, 22]. Early dcSSc patients established on MMF exhibited a lower serum protein concentration than those who were not. This may explain the better performance of the composite marker in the BIOPSY cohort than in the MODERNISE cohort, where most cases were established on MMF as the SOC. This is further corroborated by lower composite biomarker scores among early dcSSc patients established on MMF and does suggest it could act as a pharmacodynamic surrogate marker. If there is an impact of MMF or other treatments on the constituent proteins of the composite biomarker that precedes impact on mRSS itself, this may also explain the weaker association of the protein levels and composite score in the MODERNISE cohort, which includes more late-stage dcSSc cases, reflected in a greater range and higher median age. This should be explored in future studies to ensure this is a true reflection of treatment effect rather than natural disease progression.

We did not find a significant difference in treatment effect according to ANA subgroup, although this may be due to smaller sample sizes as allowed by ELISA plates. We note that there was a difference between the ATA and ARA subgroups in the longitudinal analysis of the BIOPSY cohort by normalized protein expression [13]. This warrants further exploration in larger future studies.

An important limitation is that since this was a single-centre study, we are limited in its evaluation of external validity. However, all assessments and mRSSs were performed by a single individual to ensure greater consistency of clinical assessment, treatment and sample collection and processing. The relatively small number of cases in each cohort is a major limitation and may explain why only trends of association were observed for some of the proteins that had been highly significant by Olink assay in the derivation BIOPSY cohort. This may be especially important in dissecting the impact of disease duration and background treatment that may impact on serum protein levels. Therefore, future validation studies should include more patients and may revisit the Olink proximity extension assay platform or other protein assay methodologies. The technical limitations in SPON1 measurement are relevant and warrant further study using more sensitive assay methods.

Together, these findings are supportive of the potential to develop a composite serum biomarker for skin severity in SSc. Further studies should explore the potential as a predictive or pharmacodynamic surrogate.

## Supplementary Material

rkae039_Supplementary_Data

## Data Availability

The data supporting the findings of this study are available within the article and can be made available for purposes of academic collaboration upon reasonable request to the corresponding author.
