# Supplementary material for: Testing a candidate composite serum protein marker of skin severity in systemic sclerosis
Source: Rheumatol Adv Pract. 2024 Mar 9;8(2):rkae039. doi: 10.1093/rap/rkae039 (PMC11031358; doi:10.1093/rap/rkae039)

Supplementary Table S1. Table of coefficients for multiple linear regression analysis predicting mRSS from COMP, COL4A1 and TNC.

Predictor variable	Coefficient (β)	Standard error	t-value	p-value
Intercept	9.896	2.625	3.770	0.0003*
COMP	0.01719	0.004936	3.483	0.0008*
COL4A1	-0.006481	0.02302	0.2816	0.7791
TNC	-0.002318	0.05256	0.04410	0.4105

Supplementary Figure S1. Testing and validation of COL4A1. COL4A1 concentration against mRSS, disease duration and standard of care in the BIOPSY cohort (panel A), the MODERNISE cohort (panel B) and in combined early dcSSc patients from both cohorts (panel C).

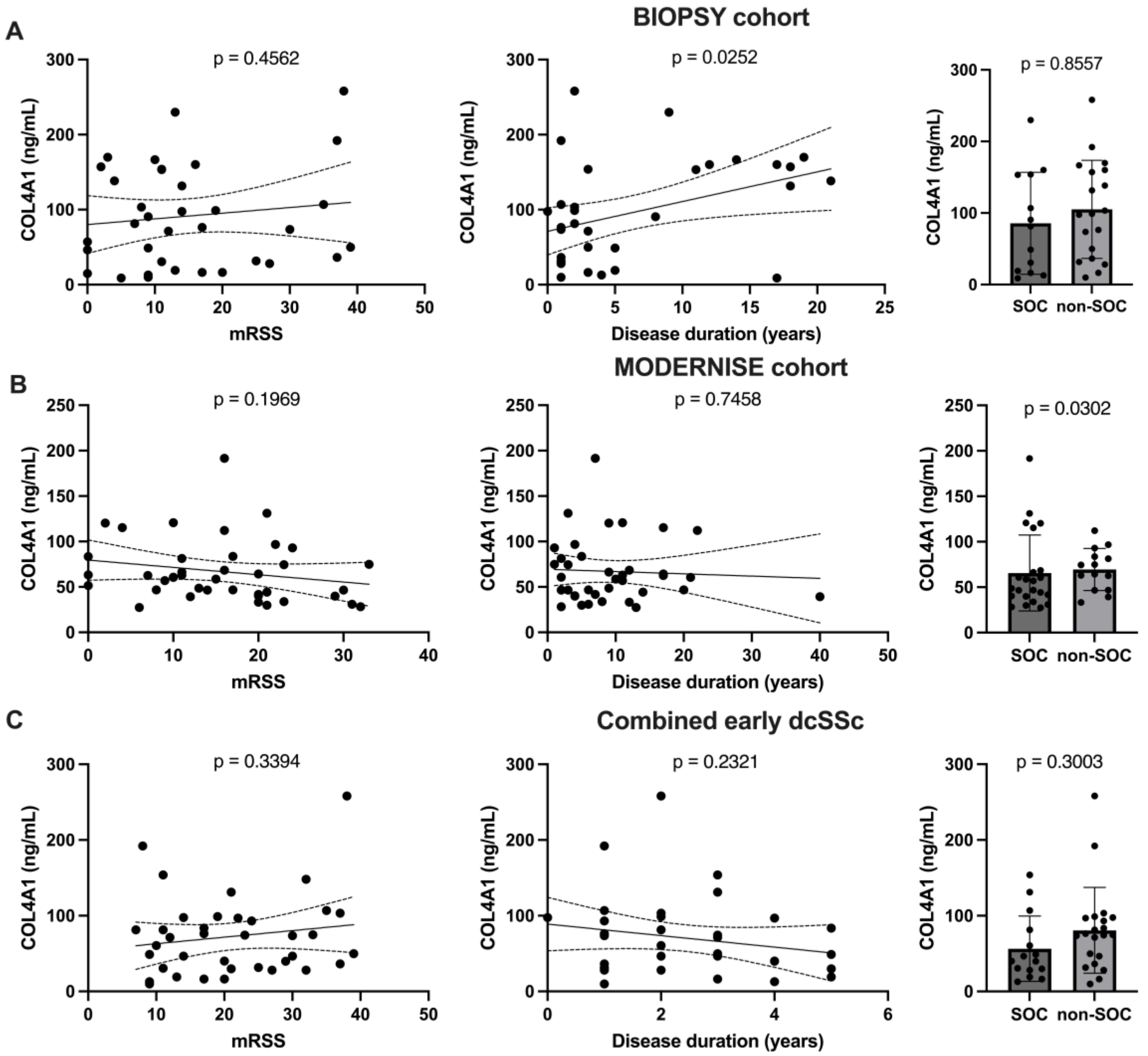

Supplementary Figure S2. Further testing and validation of COL4A1. COL4A1 concentration in the BIOPSY cohort against (A) mRSS in non-SOC and (B) disease duration in SOC patients of less than 5 years disease duration.

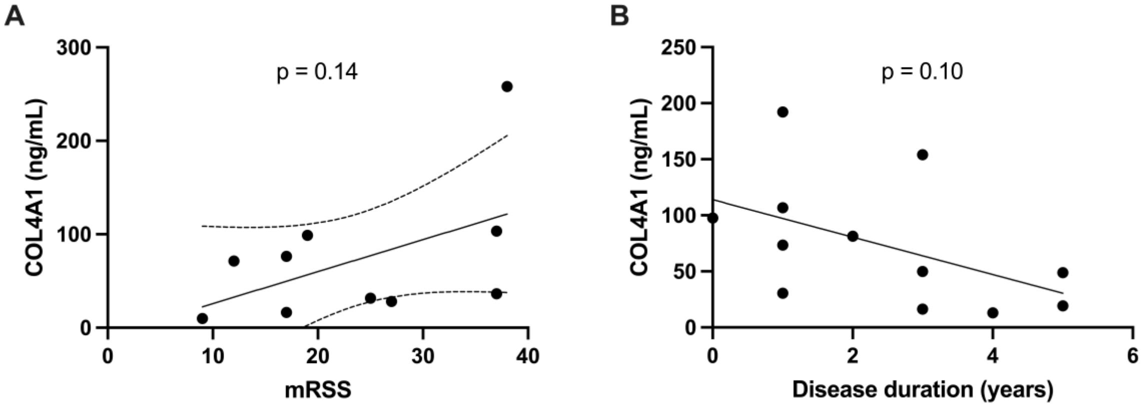

Supplementary Figure S3. Testing and validation of SPON1. SPON1 concentration against mRSS, disease duration and standard of care in the BIOPSY cohort (panel A), the MODERNISE cohort (panel B) and in combined early dcSSc patients from both cohorts (panel C).

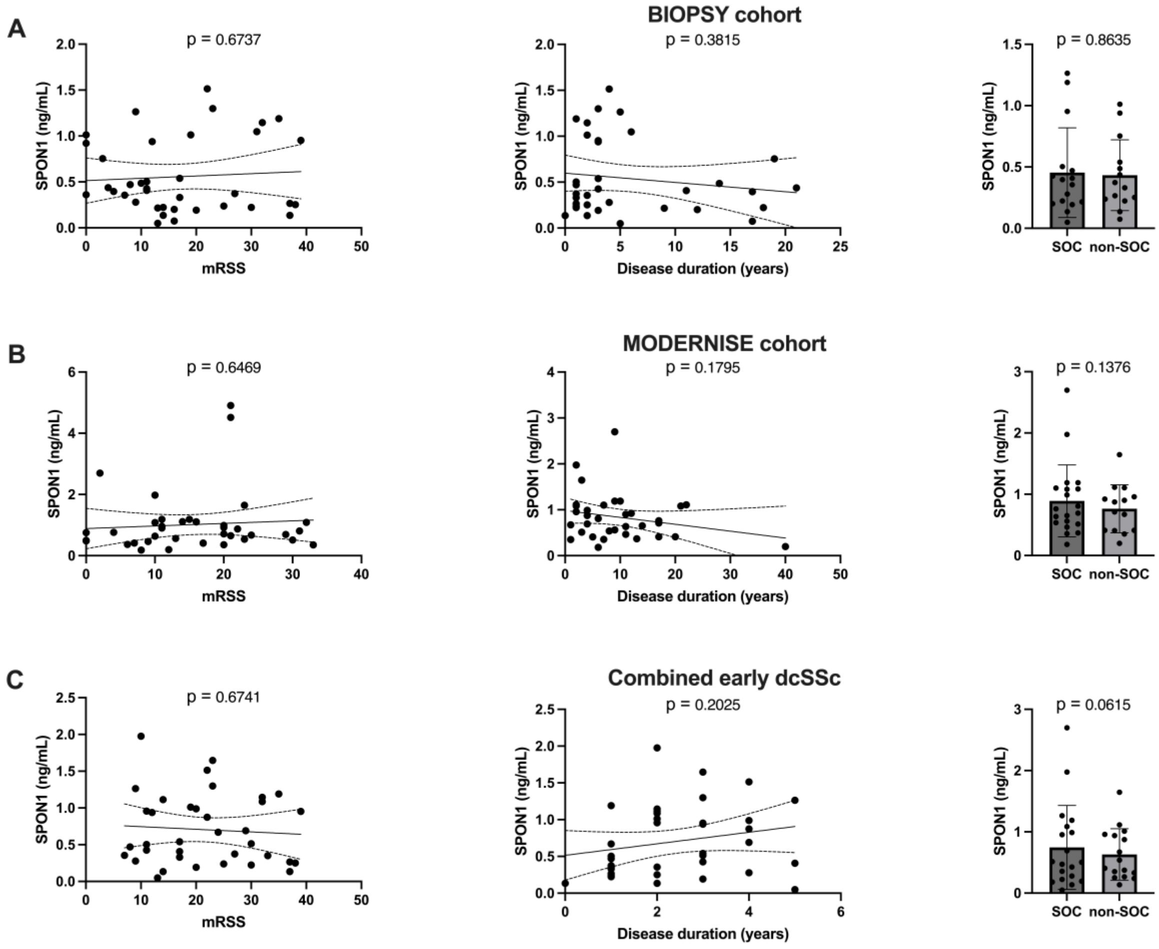

Supplementary Figure S4. Feasibility study of SPON1. Correlation of SPON1 serum concentration with (A) mRSS, (B) disease duration and (C) standard of care in the BIOPSY cohort using a different ELISA kit.

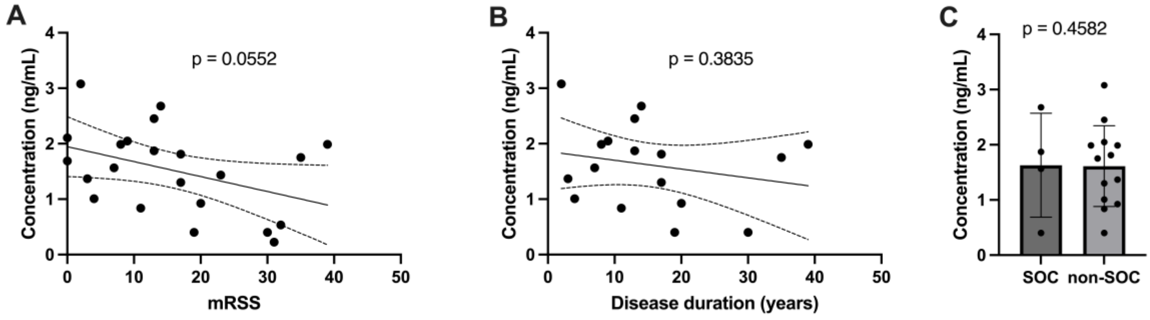

Supplementary Figure S5. Serum protein concentration by ANA subset. Serum COMP, COL4A1 and TNC concentration by MMF duration in anti-topoisomerase antibody (panel A) and anti-RNA polymerase III antibody (panel B) early diffuse cutaneous systemic sclerosis patients.

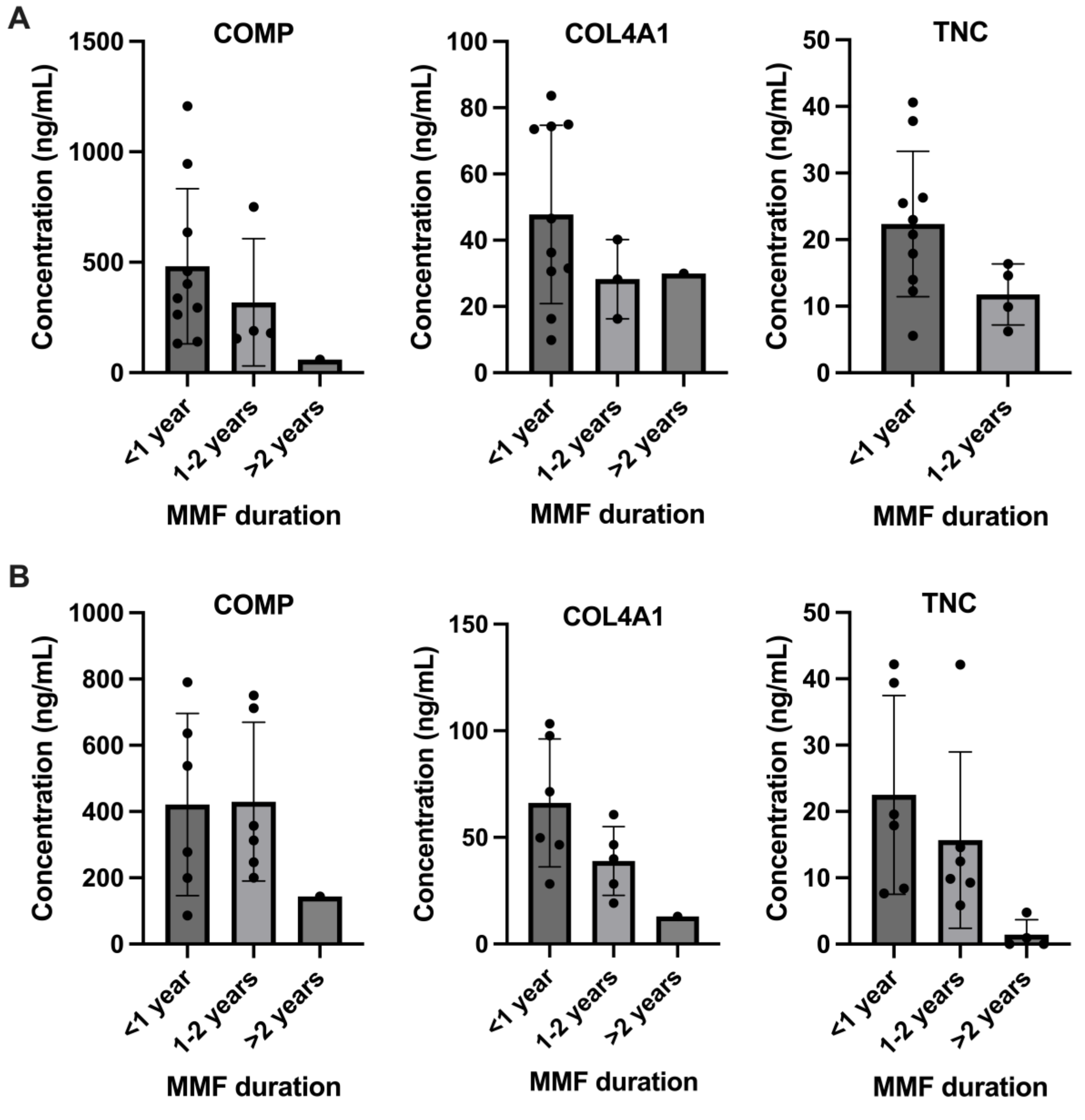

Supplement: rkae039_Supplementary_Data [file rkae039_supplementary_data.pdf]
